# Essential newborn care practices and associated factors among home delivered mothers in Damot pulasa Woreda, southern Ethiopia

**DOI:** 10.1186/s12978-018-0609-1

**Published:** 2018-09-27

**Authors:** Tesfaye Yitna Chichiabellu, Baze Mekonnen, Feleke Hailemichael Astawesegn, Birhanu Wondimeneh Demissie, Antehun Alemayehu Anjulo

**Affiliations:** 1 Department of Nursing, College of Health Science and Medicine, Wolaita Sodo University, P.O.Box: 138, Wolaita Sodo, Ethiopia; 2 Department of Nursing, School of nursing and midwifery, Addis Ababa University, Addis Ababa, Ethiopia; 3 School of Public Health, College of Medicine and Health Science, Hawassa University, Hawassa, Ethiopia; 4 Department of Nursing, College of Health Science and Medicine, Wolaita Sodo University, Sodo, Ethiopia; 5 Department of Medical Laboratory, College of Health Science and Medicine, Wolaita Sodo University, Sodo, Ethiopia

**Keywords:** Essential newborn care practice, Newborn

## Abstract

**Background:**

Globally 3.1 million children die each year in their neonatal period (first 28 days of life) according to World Health Organization (WHO) 2011 report. Half of these surprisingly occur within the first 24 h of delivery and 75% occur in the early neonatal period.

**Methods:**

A community based cross-sectional study design was carried out from March 2016 to April, 2016 in Damot Pulasa district, Wolaita zone, Southern Ethiopia to assess selected essential newborn care practices and associated factors among home delivered mothers in Damot pulasa district. Data were entered into Epi Info version 3.5.1 and exported to SPSS version 20 software for analysis. Multiple logistic analyses were done to control possible confounding variable. A *P*-value less than 0.05 was taken as a significant association.

**Result:**

The study showed that the prevalence of essential newborn care practice was 24%. Multivariate logistic regression analysis revealed that variables like ANC visit (AOR =0.213,*P* = 0.015,CI = 0.102–0.446),PNC visit (AOR = 0.209, *P* = 0.00,CI = 0.110–0.399), advice about essential newborn care practice (AOR =0.114,*P* = 0.0001, CI = 0.058–0.221),urban areas women (AOR =2,*P* = 0.042, CI = 1.024–3.693), planned pregnancy (AOR = 7, P = 0.00, CI =3.732–11.813), and knowledge about newborn danger signs (AOR = 0.277, *P* = 0.006, CI = 0.110–0.697) were the independent predictors of ENBC practices.

**Conclusion:**

Generally**,** coverage of essential newborn care practices was low. ANC visit, advice about ENBC, PNC visit, residence, planned pregnancy and knowledge about newborn danger signs were predictors of essential newborn care practice in the study area. Therefore, Health facilities should enhance linkage with health posts to increase ANC and PNC service utilization. Health extension workers should also promote and give health education about pre-lacteal feeding, early bathing, planned pregnancy, newborn danger signs and application of materials on the newborn stump.

**Electronic supplementary material:**

The online version of this article (10.1186/s12978-018-0609-1) contains supplementary material, which is available to authorized users.

## Plain English summary

Though many efforts had been made to overcome newborn mortality in Sub-Saharan Africa, it is continued to be great public health problems. Essential newborn care is a comprehensive strategy designed to improve the health of newborns through interventions before conception, during pregnancy, at and soon after birth, and in the postnatal period. Data associated with socio-demographic variables, maternal health services utilization, knowledge, counseling from a health worker, source of information, and traditional practices were collected. Even though majority of the women used boiled blade to cut the cord and tied with threads, application of butter on the umbilical stump practiced by most of the women. In addition to low coverage of initiation of breast feeding within one hour and giving colostrum, the majority, of the women in this study gave pre-lacteals. Bathing of the newborn after 24 h was practiced by the majority of the women. The level of coverage of essential newborn care practices in the district was generally low. The associated factors of essential newborn care practice were; ANC visit, advice about ENBC, PNC visit, residence, planned pregnancy and knowledge about newborn danger.

In conclusions; Health facilities should enhance linkage with health postse to increase antenatal and postnatal care service utilization. Health extension workers should also promote and give health education about pre-lacteal feeding, early bathing, planned pregnancy, newborn danger signs and application of materials on the newborn stump.

## Background

Globally 3.1 million children die each year in their neonatal period (first 28 days of life) according to World Health Organization (WHO) 2011 report. Half of these surprisingly occur within the first 24 h of delivery and 75% occur in the early neonatal period (0 to 6 days after delivery) because of preterm births, severe infections and birth asphyxia [1]. Though many efforts had been made to overcome newborn mortality in Sub-Saharan Africa, it is continued to be great public health problems. Every year 2.9 million babies die during the neonatal period [2]; it is also the time of greatest risk for stillbirths and maternal deaths [3].

One of the targets of the MDG was a two-thirds reduction in infant and child mortality by 2015; it was intended to achieve by involving skilled birth attendants, increasing immunization coverage against six vaccine preventable diseases, improving the status of women through education, and enhancing women participation in the labor force [4].

Globally, around 40 million mothers give birth at home per year without any trained health worker. Factors like lack of good quality care during labor and birth; socio-economic aspects of poverty; poor health status of women; lack of autonomy and decision making authority; and illiteracy to health system related factors like poor antenatal and obstetric care; absence of trained birth attendant; inadequate referral system; lack of transportation facilities; poor linkages between health centers and communities favored the morbidities and mortalities of pregnant women, perinatal and neonate [5]. In Ethiopia, according to Ethiopia Mini Demographic Health Survey 2014 report, only 15% of births take place at a health institution, 40% of women receive Antenatal Care (ANC) from a skilled provider, and 12% of women receive a postnatal care (PNC) within the first two days of birth [6]. This favors neonatal morbidity and mortality rates to be high in Ethiopia; around 122,000 newborns die every year and the neonatal mortality rate is 37 per 1000 live births [7, 8].

WHO recommended Essential Newborn Care (ENBC) practices to reduce the risk of the main causes of neonatal deaths in both community and facility deliveries [8]. ENBC is a comprehensive strategy designed to improve the health of newborns through interventions before conception, during pregnancy, at and soon after birth, and in the postnatal period [9].ENBC practices, as recommended by WHO, include drying (wiping) and wrapping the newborn immediately after birth, initiating skin-to-skin contact, dry cord care (not applying any potentially harmful substance to the umbilical cord), immediate initiation of breastfeeding and delayed bathing (for at least 6 h) [10].

Ethiopia government has been striving to achieve the 3rd Sustainable Development Goal (SDG3) which is to ensure healthy lives and promote well-being for all, at all ages [11]. However, the neonatal mortality rates in Damot pulasa is still remained higher than the national level; it is 38 per 1, 000 live births [12]. Thus, new innovative strategies must be developed for safe home deliveries including essential neonatal care in order to change the practice at the household level, besides devising means of proper care of the neonate in domestic settings and ensuring proper referral of those neonates who cannot be managed at home [13]. A study showed that home-based counseling strategy using volunteers and designed for scale-up can improve newborn care behaviors in rural communities [14].

Traditional Birth Attendants (TBAs), relatives, neighbors and other aged women from the community who lack the requisite knowledge of safe delivery and newborn care practices; Meanwhile, their intervention to support mothers who give birth at home is inevitable. This may increase maternal and newborn morbidity and mortality among home delivered mothers. Traditional practice like pre-lacteal feeding, avoiding of first milk and application of material on the newborn stump was practiced by the majority of study participant in the study area. Therefore, improving newborn survival is a major priority in child health today and the government sets universal sustainable development goals which state to end preventable deaths of newborns and under-five children by 2030. Therefore, this study aimed to assess selected essential newborn care practices and associated factors among home delivered mothers in Damot pulasa district.

## Methods and materials

### Study area

A community based cross-sectional study design was conducted from March 2016 to April 2016 in Damot Pulasa district, Wolaita zone, Southern Ethiopia. Damot Pulasa located at 365 Km from Addis Ababa, the capital city of Ethiopia. The population of the district was estimated to be 130,515 with an estimated number of women of reproductive age group 30,818 which is 23.6% of the total population. The town has an urban kebele and 22 rural kebeles, in terms of health facilities; there are 5 governmental health centers, 8 private clinics, 1 private pharmacy, 1 drug vender and 1urban and 22 rural health posts.

### Populations

The study population was randomly selected women of reproductive age group who had given birth at home in the past one year in Damot Pulasa district which encompasses 450 women who participated in the study. Those mothers who had given live birth at home within one year preceding the data collection date included in the study. The source population was list of households who had women’s in the reproductive age and who had given birth at home in Damot pulasa district.

### Sample size determination

The required sample size was determined by using single population proportion formula by taking 23% of expected prevalence for essential newborn care practice [8], assuming 5% margin of error and 95% confidence level, design effect of 1.5 and 10% for non-response rate. The calculated sample size was 450.

### Sampling technique and procedures

Cluster multi-stage sampling technique was employed for the Selection of the sampling units. In the district, there are 22 rural and an urban kebeles. From 22 rural kebeles10 were selected by simple random sampling. The total sample size was allocated for each selected kebeles proportionally to the number of households within each kebele. Then systematic sampling technique was used to select a household where participant exist. The index case was selected and interviewed using lottery method when more than one eligible respondent present in a house.

### Data collection tools and procedures

Data associated with socio-demographic variables, maternal health services utilization, knowledge, counseling from a health worker, source of information, and traditional practices were collected using interviewer administered questionnaire adapted from similar studies [8, 14, 18] (Additional file 1). The data were collected by B.Sc. nurses who are fluent speakers of the local languages.

### Data processing and analysis

Data was checked visually for completeness, and then coded and entered in to Epi Info version 3.5.1 and exported in to Statistical Program Social Science (SPSS) version 20 software for analysis. Binary and multiple logistic regressions were run to assess the associations of various factors with essential newborn care practice. The results were presented in the form of tables, figures and summary statistics. A *P*-value less than 0.05 was taken as a significant association.

## Results

### Socio-demographiccharacterstics

In this study, a total of 450 women have participated and the response rate was 100%. In terms of religion, majority of the respondents were protestant, which accounts 238 (52.9%) and 434 (96.4%) were Wolaita in ethnicity. One hundred ninety-five (43.3%) were illiterate and 310 (68.9%) were housewife. With regard to marital status and place of residence, 444 (98.7%) were married and 393 (87.3%) were rural dweller (Table 1).

**Table 1:** Socio-demographic characteristics of the respondent in Damot pulasa district, Wolaita Zone, Southern Ethiopia, 2016

Variable	Frequency (*n* = 450)	Percentage (%)
Religion		
Protestant	238	52.9
Catholic	124	27.6
Orthodox	76	16.9
Muslim	12	2.7
Educational status		
No education	195	43.3
Primary level	175	38.9
Secondary level	67	14.9
Higher education	13	2.9
Ethnic group		
Wolaita	434	96.4
Gammo	8	1.8
Amhara	7	1.6
Gurage	1	.2
Occupation		
Housewife	310	68.9
Farmer	13	2.9
Merchant/Trade	100	22.2
Daily labor	27	6.0
Marital status		
Married	444	98.7
Widowed	6	1.3
Residence		
Urban	57	12.7
Rural	393	87.3
Age at current pregnancy		
< 20 years	2	0.4
20–34 years	364	80.9
34–49 years	84	18.7
Planned pregnancy		
Yes	67	14.9
No	383	85.1
Parity		
1	44	12.8
2–4	194	56.6
> = 5	105	30.6

### Maternal health services

A total of 364 (80.9%) of respondents belonged to the age group 19–41 years and the mean age of respondents was 30.8 (± 4.05). Majority of the study subjects conceived their last baby unintentionally, which accounts 383 (85.1%). Thirty two (7.1%) received at least one ANC visit. From all mothers, 363 (80.7%) prepared themselves for birth. From the total study subjects, 120 (26.7%) utilized PNC service and from these mothers, 35 (29%) utilized the service within 7-41 days (Table 2).

**Table 2:** Maternal health services of respondents, in Damot pulasa district, Wolaita Zone, Southern, Ethiopia, 2016

Variable	Frequency (n = 450)	Percentage (%)
Receive ANC		
Yes	32	7.1
No	418	92.9
Number of ANC visit		
Once	13	40.6
Twice	10	31.25
Three times	6	18.75
Four times	3	9.4
Advice about ENBC		
Yes	32	7.1
No	418	92.9
Preparation for delivery		
Yes	363	80.7
No	87	19.3
Receive PNC		
Yes	120	26.7
No	330	73.3
Time for frequency of PNC		
Less than 4 h	12	10
4–23 h	20	16.7
1–2 days	27	22.5
3–6 days	26	21
7–41 days	35	29

### Health service availability

**Fig. 1:**
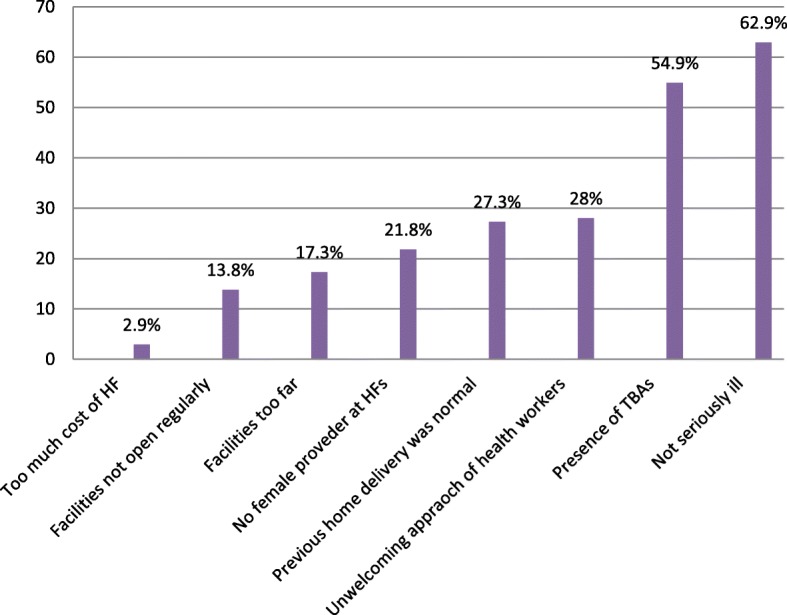
Reasons of women not delivered at health facilities, in Damot pulasa district, Wolaita Zone, Southern Ethiopia, 2016

Concerning health service availability, 318 (70.7%) mothers had health facilities (health post)in the nearby site. Home delivered mothers mentioned the following reasons why they gave birth at home; Two hundred eighty-three (62.9%) “Not seriously ill”, 247 (54.9%) “Had TBAs”, 126 (28%) “Unwelcoming of health workers approach” and 123 (27.3%) “An experience of safe home delivery before” Moreover, the majority of women participated in this study, 356 (79.1%) decided to deliver at home by themselves (Table 3) (Fig. 1).

**Table 3:** Health service utilization of respondents, in Damot pulasa district, Wolaita Zone, Southern Ethiopia, 2016

Variable	Frequency (n = 450)	Percentage (%)
Availability of HF		
Yes	450	100
Type of HF		
Health post	318	70.7
Health center	132	29.3
HF provide delivery		
Yes	227	50.4
No	179	39.8
I don’t know	44	9.8
Decision for place of birth		
Self	356	79.1
Husband	77	17.1
Relatives	17	3.8

### Knowledge of the respondents

**Fig. 2:**
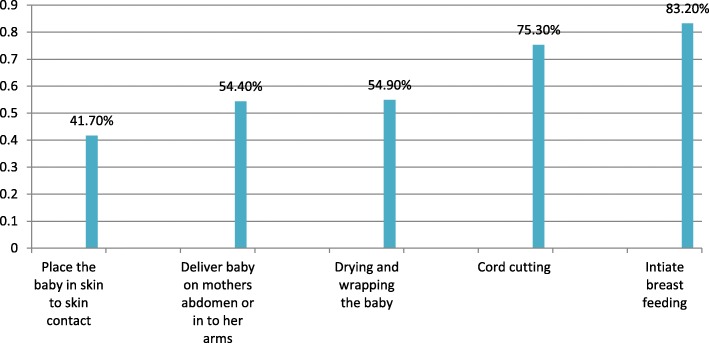
Knowledge of women about ENBC practices in Damot pulasa district, Wolaita Zone, Southern Ethiopia, 2016

**Fig. 3:**
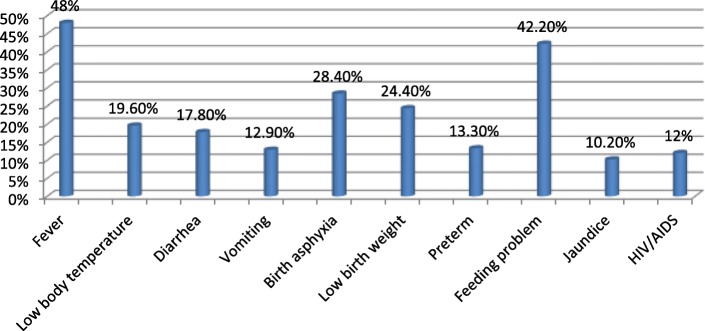
Knowledge of women on newborn danger signs in Damot pulasa district, Wolaita Zone, Southern Ethiopia, 2016

From the total study subjects, 417 (92.7%) of the women had information about newborn care. Among 415 (92.2%) women who had information about when to start breastfeeding, 141 (34%) mothers started breastfeeding within the first one hour of birth. From all mothers, 390 (86.7%) of them had knowledge about colostrum and 262 (67%) mothers mentioned the importance of colostrum. Four hundred six (90.2%) of women told that it is possible to expose the neonate for morning sunlight; In addition to this, 432 (96%) of women mentioned that, exposing the neonate for vaccination has no problem (Table 4) (Figs. 2 and 3).

**Table 4:** Knowledge of the respondents, in Damot pulasa district, Wolaita Zone, Southern Ethiopia, 2016

Variable	Frequency (n = 450)	Percentage (%)
Information on newborn care		
Yes	417	92.7
No	33	7.3
Information to start breastfeeding		
Yes	415	92.2
No	35	7.8
Time to start breastfeeding		
First one hour	141	34.0
After one hour	274	66.0
Knowledge on first milk		
Yes	390	86.7
No	60	13.3
Advantage of first milk		
Advantageous	262	67.0
Disadvantageous	129	33.0
Expose neonate for morning sunlight		
Yes	406	90.2
No	44	9.8
Expose neonate for vaccination		
Yes	432	96.0
No	18	4.0
Information when to bath the neonate		
Yes	436	96.9
No	14	3.1
Time of bathing		
First 24 h	303	69.5
After 24 h	133	30.5
Knowledge about neonatal problems		
Good knowledge	87	19.3
Poor knowledge	363	80.7

### Newborn care practice of the respondent

From all mothers, 408 (90.7%) remembered where they positioned the neonate immediately after delivery. Of all mothers, 259 (63.5%) put their newborn baby on their abdomen immediately after delivery (Table 5).

**Table 5:** Newborn care practices of respondents, in Damot pulasa district, Wolaita Zone, Southern Ethiopia, 2016

Variable	Frequency (n = 450)	Percentage (%)
Position the neonate		
Yes	408	90.7
No	42	9.3
Place of positioning the neonate		
On the mother’s abdomen	259	63.5
Near the delivery surface	86	21.1
On another bed separately	52	12.7
Transferred to father/relatives	9	2.2
I don’t remember	2	.5
Dry/wrapping the neonate		
Yes	362	80.4
No	88	19.6
Time of dry/wrap the neonate		
Before delivery of placenta	190	52.2
Immediately after delivery of placenta	169	46.4
I did not remember	5	1.4
Material used for dry/wrap the neonate		
Pre-prepared towel	166	45.6
Piece of blanket/Gabi	72	19.8
Available material	126	34.6
Material used to cut the cord		
Boiled /un-boiled new razor blade	434	96.4
Used razor blade	16	3.6
Remember material used to tie the cord		
Yes	442	98.2
No	8	1.8
Material used to tie the cord		
Thread	442	100
Apply material after cord cutting		
Yes	288	64.0
No	162	36.0
Type of material applied on the cord		
Butter	288	100
Initiate exclusive breastfeeding		
Yes	226	50.2
No	224	49.8
Time of initiating exclusive breastfeeding		
First one hour	206	45.8
After one hour	244	54.2
Give pre lacteals		
Yes	224	49.8
No	226	50.2
Pre-lacteals given		
Water	218	97.3
Butter	6	2.7
Give first milk		
Yes	223	49.6
No	227	50.4
Frequency of breastfeeding		
< 8 times	203	45.1
> = 8 times	247	54.9
Remember time of bathing		
Yes	442	98.2
No	8	1.8
Time of bathing		
First 24 h	156	34.7
After 24 h	294	65.3
Skin to skin contact		
Yes	249	55.3
No	201	44.7

### Safe cord cutting

Almost all mothers, 434 (96.4%) used boiled new razor blade in order to cut their newborn baby and 288 (64%) study subjects applied butter on the cord after the cord was cut.

### Initiation of early exclusive breastfeeding

From the total study subjects, 206 (45.8%) initiated breastfeeding within an hour of birth. From the total of 224 (49.8%) women who gave pre-lacteals, 218 (97.3%) gave water and 6 (2.7%) gave butter. Two hundred twenty three (49.6%) of the respondents gave first milk to the newborn and 247 (54.9%) mothers fed their breast greater than or equal to eight times.

### Thermal care (bathing time)

From all mothers, 362 (80.4%) mothers dried/wrapped the newborn baby. Of whom 190 (52.2%) dried/wrapped the newborn before delivery of the placenta. One hundred sixty-six (45.6%) mothers used a pre-prepared towel to dry/wrap up the newborn. About 294 (65.3%) of them bathed the newborn after 24 h. The majority of study subjects, 249 (55.3%) mothers made skin to skin contact of mother and newborn.

### The prevalence of essential newborn care practices

**Fig. 4:**
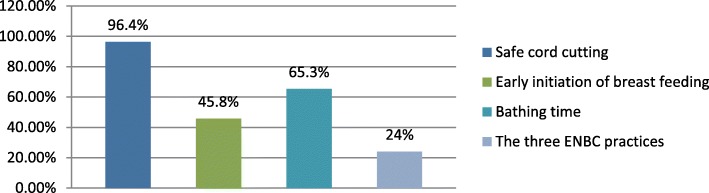
Distribution of the three essential newborn care practices, in Damot pulasa district, Wolaita Zone, Southern Ethiopia, 2016

The prevalence of cord cutting, initiation of breastfeeding and thermal care practices were studied in this study. This study revealed that the prevalence of cord cutting, initiation of breastfeeding and thermal care practices were 434 (96.4%), 206 (45.8%) and 294 (65.3%) respectively (Fig. 4).

### Associated factors of essential newborn care practices

In order to determine the association of independent variables with essential newborn care practices both bivariate and multivariate analysis were used. Variables that showed association with the outcome variables in the bivariate analysis were selected for multivariate analysis.

Crude analysis revealed that variables like ANC visit (COR 0.213, 95% CI: 0.102–0.446), advice about ENBC (COR 0.166, 95% CI: 0.078–0.354), PNC visit (COR0.135,95% CI:0.083–0.217), place of residence (COR2.244, 95% CI:1.251–4.025), planned pregnancy (COR 6.863, 95% CI: 3.943–11.943), birth preparedness (COR 3.511, 95% CI: 1.635–7.541), knowledge about newborn danger signs (COR 5.276, 95% CI: 2.232–12.471), and knowledge about newborn care (COR 1.892, 95% CI: 1.223–2.928) were felt to be the key predictors of essential newborn care practice and were used to form multivariable logistic regression analysis (Table 6).

**Table 6:** Factors associated with the three essential newborn care practices by bivariate and multiple logistic analyses in Damot pulasa district, Wolaita Zone, Southern Ethiopia, 2016

Variable	ENBCP		COR	AOR
	Yes (%)	No (%)		
Receive ANC				
Yes	18	14	1	1
No	90	328	0.213(0.102–0.446)	0.264(0.090–0.773)*
Advice about ENBC				
Yes	19	13	1	1
No	61	252	0.166(0.078–0.354)	0.114(0.058–0.221)*
Receive PNC				
Yes	64	56	1	1
No	44	286	0.135(0.083–0.217)	0.209(0.110–0.399)*
Residence				
Urban	22	35	2.244(1.251–4.025)	2(1.024–3.693)*
Rural	86	307	1	1
Planned pregnancy				
Yes	40	27	6.863(3.943–11.943)	7(3.732–11.813)*
No	68	315	1	1
Birth preparedness				
Yes	8	75	3.511(1.635–7.541)	0.467(0.200–1.087)
No	100	267	1	1
Knowledge about newborn danger signs				
Good knowledge	6	102	5.276(2.232–12.471)	0.277(0.110–0.697)*
Poor knowledge	81	261	1	1
Knowledge about newborn care				
Good knowledge	49	209	1.892(1.223–2.928)	0.760(0.460–1.257)
Poor knowledge	59	133	1	1

Multivariate logistic regression was done for variables that had statistically significant association with essential newborn care practice in crude analysis. Multivariate logistic regression analysis revealed that variables like ANC visit (AOR 0.264, 95% CI:0.090–0.773), advice about essential newborn care practice (AOR 0.114, 95% CI:0.058–0.221), PNC visit (AOR 0.209, 95% CI:0.110–0.399), place of residence (AOR 2, 95% CI:1.024–3.693), planned pregnancy (AOR 7, 95% CI:3.732–11.813), and knowledge about newborn danger signs (AOR 0.277, 95% CI:0.110–0.697) were the independent predictors of essential newborn care practice after controlling the potential confounders (Table 6).

## Discussion

Generally, in this study the coverage of essential newborn care practice was low. Even though majority of the women used boiled blade to cut the cord (96.4%) and tied with threads (98.2%), application of butter on the umbilical stump (64%) of the women practiced. I n addition to low coverage of initiation of breast feeding within one hour (45.8%) and giving colostrums (49.6%),the majority, (49.8%) of the women in this study gave pre-lacteals. Bathing of the newborn after 24 h was practiced by the majority (65.3%) of the women.

The prevalence of ENBC practice was 24% which was higher than the research done in Awebel district East Gojam Zone [8] which was 23.1% but which was much lower than the study conducted in Northwest Ethiopia, Mandura district [15] which was 41%. Cord cutting was practiced by the majority 96.4% of the women, using new blade, which was much higher than the study conducted in, Nawalparasi district of Nepal (48.31%) [16], Northern Ghana which revealed 90.8% [17], Sub urban areas of western Nigeria (90.3%) [18], study conducted in Northwest Ethiopia, Mandura district was (59.8%) [15],and the study conducted in four regions of Ethiopia which was 88.3% [6], the reason for this might be good awareness and custom followed in the study area but the finding was in line with the study conducted at Awebel district, East Gojam of Ethiopia (97.6%) [8]. Majority of the study participants (98.2%) the cord was tied with thread which was higher than the study conducted in the four regions of Ethiopia (48.5%) [6], this might be due to awareness in the study community. Even though majority of the women used boiled blade to cut the cord and tied with threads, application of butter on the umbilical stump (64%) of the women practiced in the study area which is higher than the study conducted in Northern Ghana (14.4%) and the study conducted in Northwest Ethiopia Mandura district was (18.18%) [15, 17] but which was lower than the study conducted in the four regions of Ethiopia (88.3%) [6].

Initiation of breastfeeding within one hour in the study area was 45.8% which was higher than the study conducted in rural Bangladesh (40%), East Gojam of Ethiopia (41.6%). This finding was not incongruent with the study conducted in India (65%), Nepal (51.3%), Northern Ghana (80%), Eastern Uganda (50%), Western Nigeria (65.3%), four regions of Ethiopia (52.1%), Northwest Ethiopia and Southwest Ethiopia (50%) [6, 15–21] respectively. The Majority, 49.8% of the women in this study gave pre-lacteals. The finding was higher as compared to study conducted in the four regions of Ethiopia (12.4%) gave pre-lacteals [6], but lower than the study conducted in East Gojam of Ethiopia Awebel district, 11.2% gave pre-lacteals [8]. The reason might be traditional beliefs of the community. Breastfeeding of the first milk (colostrum) was given (49.6%) of the women in the study area. This is lower than a case study of tribal women, Gujarat (63%) [22]. The reason for this was (33%) of the respondent believed that first milk was disadvantageous and from this (31%) believed that it would cause diarrhea,(60.5%) constipation and (58.9%) believed that it would decrease the growth of the newborn.

Bathing of the newborn after 24 h was practiced by the majority (65.3%) of the women in the study area which was in line with the study conducted in East Gojam of Ethiopia, Awebel district (65.6%) [8]. But this finding was lower than study conducted in Northern Ghana (93.6%), Rural Nepal (72.2%), South Sudan (99%), Easter Uganda (100%), Western Nigeria (98.2%), study conducted in four regions of Ethiopia (74.7%) [6, 16–18, 20, 23].

In this study women who didn’t get ANC visit were 73.6% less likely to practiced essential newborn care practice as compared to those who initiated ANC visit (AOR =0.213,*P* = 0.015,CI = 0.102–0.446), which is supported by the study conducted in Northern Ghana which suggested that women who initiated ANC visit were two times more likely to practiced essential newborn care practice as compared to women who initiated ANC visit late [17]. This might be due to women who attended ANC have the chance of getting information about the components and the importance of newborn care practice from health care providers.

The finding of this study also showed that women who didn’t get PNC visit early were 79% less likely practiced ENBC when compared to women who didn’t get immediate PNC visit (AOR = 0.209, *P* = 0.00,CI = 0.110–0.399). This finding was supported by the study conducted in rural communities of Awebel district, East Gojam of Ethiopia, which stated that immediate PNC visit was statistically significant with ENBC practice of women and those women who had got immediate PNC visit after delivery were 3.2 times more likely to practice ENBC when compared with those who had not got immediate PNC after delivery [8]. This could be health extension workers and community health workers might gave proper advice about essential newborn care practice.

Those mothers who had got ENBC advice during ANC visit or other meetings were 83.4% more likely practiced ENBC practice as compared to women who did not got the advice (AOR =0.114, *P* = 0.0001, CI = 0.058–0.221). It was supported by study done, Awebel district which showed that women who had got advice about ENBC practices during monthly pregnant mothers’ group meeting were 4.8 times more likely to practice ENBC as compared with those women who had not got advice about ENBC practices during monthly meeting [8]. The reason could be the health care providers could discuss about essential newborn care practice during ANC visit.

In this study, urban areas women were two times more likely practiced ENBC practice when compared to rural areas women (AOR =2, *P* = 0.042, CI = 1.024–3.693). The finding was supported by a study conducted in Mandura district which stated that women in urban areas were three times more likely to have good newborn care practices as compared to rural areas [15]. This might be due to accessibilities of health service and good knowledge secondary to better educational status of urban women when compared to rural areas women.

Those women who planned there pregnancy were seven times more likely practiced newborn care when compared to women who did not plan their pregnancy (AOR = 7, *P* = 0.00, CI =3.732–11.813). The reason for this could be women who had planned pregnancy might be more likely to use maternal and child health services.

The study showed that those women who were knowledge about newborn danger signs practiced ENBC 72% more likely when compared to women who had poor knowledge about newborn danger signs (AOR = 0.277, *P* = 0.006, CI = 0.110–0.697). This finding was supported by the study conducted in rural areas of Northern Ghana which states that women who could mention at least four danger signs of the neonates were four times more likely to give good neonatal feeding to their babies [17]. This could be most of the women in the sample may not have adequate knowledge about newborn care. This might be due to majority of the women did not get an adequate message about newborn care during antenatal care follow up. Findings in this study should be interpreted in the light of the inherent limitations of the study. Recall bias was a possibility since the women were inquired about events which occurred during a two year period. However, the questioning was focused on the most recent experiences of essential newborn care practices in order to minimize this possibility.

## Conclusions

In this study, the level of coverage of essential newborn care practices in the district was generally low. Traditional practice like: pre-lacteal feeding, avoiding of first milk and application of material on the newborn stump were practiced by majority of study participant in the study area. This finding also revealed that most essential newborn interventions were not reaching the newborns. ANC visit, advice about ENBC, PNC visit, residence, planned pregnancy and knowledge about newborn danger signs were predictors of essential newborn care practice in the study area. Therefore Damot pulasa district health office should promote strong community based behavior change communication on the importance of ENBC practices to change the poor ENBC practices in the study area. Health facilities should enhance linkage with health posts to increase ANC and PNC service utilization. Health extension workers should promote and give health education about pre-lacteal feeding, early bathing, planned pregnancy, newborn dander signs and application of materials on the newborn stump.

## Additional file

Additional file 1: Annex II: English version questionnaire. (DOCX 29 kb)

## Abbreviations

ANC: Anti Natal Care
EDHS: Ethiopian Demographic and Health Survey
ENBC: Essential Newborn Care
MDG: Millennium Development Goal
NMR: Neonatal Mortality Rate
PNC: Post Natal Care
SDG: Sustainable Development Goal
SPSS: Statistical Program Social Science
