# Supplementary material for: Essential newborn care practices and associated factors among home delivered mothers in Damot pulasa Woreda, southern Ethiopia
Source: Reprod Health. 2018 Sep 27;15:162. doi: 10.1186/s12978-018-0609-1 (PMC6161384; doi:10.1186/s12978-018-0609-1)
Supplement: Supplementary file 1 — Annex II: English version questionnaire. (DOCX 29 kb) [file 12978_2018_609_MOESM1_ESM.docx]

## ANNEX II: ENGLISH VERSION QUESTIONNAIRE

Questionnaire code________ Kebele ________House number _________

Part 1: socio-demographic and socioeconomic variables

| **№** | **Questions** | **Responses** | **Skip** |
| --- | --- | --- | --- |
| 101 | To which religion do you belong? | Orthodox Christian …………  Muslim …………………….  Protestant ……………………  Catholic ………………………  Other, specify ………………… |  |
| 102 | What is the highest grade you completed? | No education ……………….  Primary level ……………………  Secondary level.............................  Higher education ………………... |  |
| 103 | To which ethnic group do you belong? | Wolaita……………………………  Gammo……………………………  Amhara……………………………  Oromo ……………………………  Gurage ……………………………  Other, specify…………………… |  |
| 104 | What is your occupation? | Housewife……………………  Farmer……………………  Merchant/Trade………………  Government employee……  Daily labor……  Other (specify) ……………… |  |
| 105 | What is your current marital status? | Single…………………………  Married………………………  Divorced……………………  Widowed……………………  Other (specify)……………… |  |
| 106 | What is your residence? | Urban………………  Rural……………… |  |

Part 2: Maternal health services (obstetric factors)

| № | Questions | Responses | To skip |
| --- | --- | --- | --- |
| 201 | How old were you at your 1st pregnancy in completed years? | ……………………………. |  |
| 202 | How old were you when you were pregnant this child? | <20…………………………….  20-34…………………………  35-49…………………………… |  |
| 203 | Was the pregnancy planned? | Yes …………………………….  No……………………………… |  |
| 204 | Did you receive antenatal care when you were pregnant this child? | Yes ………………………….  No……………………………… | If no skip to 207 |
| 205 | How many times did you visit ANC when you were pregnant this child? | Once………………………………  Twice……………………………  Thrice……………………………  Four times………………………  Other (specify) ………………… |  |
| 206 | During antenatal visit, have you ever been informed /advised about new born care for the babies? | Yes………………………………  No……………………………… |  |
| 207 | During your pregnancy of this child did you make any preparations for your delivery? | Yes………………………………  No……………………………… | If no skip to 209 |
| 208 | What preparations did you make for the delivery? (More than one answer is possible) | Financial…………………………  Transport…………………………  Food………………………………  Identification of skilled birth attendant.…………………………  Identification of facility…………  Clean delivery kit………………  Clothes for new born……………  Other (specify) ………………… |  |
| 209 | According to your birth order, where does this baby belong? | 1 ……………………  2 ……………………  3 ………………………  4 …………………………  5 …………………………  6+………………………… |  |
| 210 | Who attended during this baby delivery? | No assistant……………………  Nurse/midwife nurse…………  TBA……………………………  Health extension worker………  Relative/friend…………………  Other (specify) ……………… |  |
| 211 | Did you use home delivery kit during last delivery? | Yes……………………………  No…………………………… | If no skip to213 |
| 212 | If yes, was the instrument clean? | Yes……………………………  No…………………………… |  |
| 213 | Did you receive post-natal care after the delivery of this child? | Yes……………………………  No…………………………… | If no skip to 301 |
| 214 | If yes, when did you attend? | Less than 4 hours………………  4-23 hours……………………  1-2 days………………………  3-6 days………………………  7-41 days……………………… |  |

**Part 3: Health service utilization**

| **№** | **Questions** | **Responses** | **Skip** |
| --- | --- | --- | --- |
| 301 | Is there health facility in your vicinity? | Yes……………………………  No……………………………… | If no skip to 304 |
| 302 | What type of health facility is it? | Health post……………………  Health center…………………  Hospital ---------------------  Private clinic …………………  Other, specify………………… |  |
| 303 | Does the health facility provide delivery service? | Yes ……………………………  No………………………………  I do not know……………… |  |
| 304 | What were the reasons you would not delivered at health facility? | Too much cost of HFs…………  Facilities not open regularly……  Facility too far…………………  Poor quality service of HFs……  No female provider at HFs……  Husband will not allow………  Unwelcoming approach  Of health workers…....................  Presence of TBAs ……………  Previous Home delivery was normal……………………  I was told that my Pregnancy is normal……………….. ………..  Not seriously ill………………  Culturally prohibited…………  Presence of traditional healer…  Other, specify … |  |
| 305 | Who decides place for your child birth? | Self …………………… …  Husband ………………………  Relatives ………………………  Religious leader ………………  Other, specify ….……………… |  |

**Part 4: knowledge questions on newborn care**

| **№** | **Questions** | **Response** | **Skip** |
| --- | --- | --- | --- |
| 401 | Do you have information about immediate newborn care practice? | Yes…………………… …  No…………………… … | If no skip to Q404 |
| 402 | If yes, mention the source of information about newborn care(more than one answer is possible) | Family/relatives……………  Neighbors…………………  Health professional…………  TV/Radio…………………  News paper/magazines……  Others (specify) ………… |  |
| 403 | What type of immediate newborn care do you know? (More than one answer is possible) | Deliver baby on to mother’s abdomen or into her arms…  Cutting cord………………  Drying and wrapping the baby……………………  Place the baby in skin to skin contact  Initiate breast feeding……  Other (specify)…………… |  |
| 404 | Do you have information about when to start breast feeding after birth? | Yes……………………  No…………………… … |  |
| 405 | Did you mention the time when should breast feeding be started | …………hrs |  |
| 406 | Do you know about the first breast milk? | Yes…………………… …  No…………………… … | If no skip to Q409 |
| 407 | If yes, is the first milk advantageous or disadvantageous? | 1. Advantageous……………  2. Disadvantageous……… | If 1 skip to 409 |
| 408 | If disadvantageous, mention the disadvantages?  (more than one answer is possible) | Causes diarrhea……………  Causes constipation………  Decrease growth…………  Other (specify)…………… |  |
| 409 | Is exposing the neonates for morning sunlight important? | Yes ……………………  No …………………… | If yes, skip to Q411 |
| 410 | If no, mention the reasons?  (more than one answer is possible) | Causes eye problem………  Causes physical flaccid……  Others (specify)…………… |  |
| 411 | Is it possible to expose the neonate for vaccination? | Yes ……………………  No…………………… … | If yes skip to Q413 |
| 412 | If no, mention the reasons(more than one answer is possible) | Culture……………………  Lack of health facility……  Lack of knowledge………  Bad previous experience…  Individual preference………  Other (specify)…………… |  |
| 413 | Do you have information when to bath the neonate immediately after birth? | Yes……………………  No…………………… … | If no skip to 415 |
| 414 | When did the new born should be bathed, mention time? | …………hr |  |
| 415 | Do you have information about neonatal problems? | Yes……………………  No…………………… … | If no skip to Q501 |
| 416 | What neonatalhealth problems do you know?  (more than one answer is possible) | Fever……………………  Low body temperature……  Diarrhea…………………  Vomiting…………………  Birth asphyxia /breathing difficulty………………  Low birth weight………  Preterm………………  Feeding problem………  Jaundice………………  HIV infection……………  Others (specify)………… |  |

**Part 5: newborn care practice questions**

| № | **Question** | **Response** | **Skip** |
| --- | --- | --- | --- |
| 501 | Did you remember where you positioned the neonate/s immediately after delivery? | Yes…………………… ………  No…………………… ……… | IIf no skip to 503 |
| 502 | If you remember, where you positioned the neonate/s immediately after delivery? | On the mother’s abdomen………  Near the delivery surface………  On another bed separately………  Transferred to father/relatives……  I don’t remember………………  Others (specify) ……………… |  |
| 503 | Did you dry/wrapping the neonate after birth? | Yes…………………… ………  No…………………… ……… | If no skip to 506 |
| 504 | If you dry/wrap the neonate, when did you dry/wrap? | Before delivery of placenta………  Immediately after delivery of placenta……………………  I did not remember……………  Other specify…….. |  |
| 505 | What material did you use to dry the neonate/s’ body? | Pre-prepared towel………………  Piece of blanket/Gabi…………  Available material………………  I don’t remember…………………  Other (specify) ………………… |  |
| 506 | Did you remember the material used to cut the cord? | Yes…………………… ………  No…………………… ……… | If no skip to 508 |
| 507 | If you remember, what material was used to cut the cord? | Un-boiled new razor blade………  Boiled new razor blade………  Un-boiled used razor blade………  Boiled used razor blade…………  Available sharp instrument………  Others (specify) ……………… |  |
| 508 | Did you remember the material you used to tie the cord? | Yes…………………… ………  No…………………… ……… | If no skip to Q510 |
| 509 | If yes, what was the material you used? | Thread…………………… …  Cord tie…………………… …  Cord clamp……………………  Others (specify) ………………… |  |
| 510 | Did you apply any material on the cord after cutting? | Yes…………………… ………  No…………………… ……… | If no skip to Q512 |
| 511 | If yes, mention type of material applied on the cord | Cow dung…………………… …  Dust…………………… ………  Butter…………………… ………  Ash…………………… ………  Others (specify) ………………… |  |
| 512 | Did you initiate exclusive breast feeding immediately after birth? | Yes…………………… ………  No…………………… ……… | If no skip to Q514 |
| 513 | When did you initiate exclusive breast feeding? | First one hour……………………  After one hour…………………… |  |
| 514 | Did you give additional diet (pre-lacteal) within 28 days | Yes…………………… ………  No…………………… ……… | If no skip to Q516 |
| 515 | If yes, what did you give for the neonate? | Water…………………… ………  Butter…………………… ………  Cow milk/ commercial milk formula…………………… …  Holy water……………………  Other (specify) ………………… |  |
| 516 | Did you give the 1^st^ milk for your neonate? | Yes …………………… ………  No…………………… ……… |  |
| 517 | How often did you breast feed the neonate per day? | < 8 times…………………… …  >= 8 times…………………… … |  |
| 518 | Did you remember the time you bathed your neonate? | Yes…………………… ………  No…………………… ……… | If no skip to 520 |
| 519 | How long after birth was your baby bathed for the first time? | First 24 hours……………………  After 24 hours…………………… |  |
| 520 | Did you keep your neonate immediate skin to skin contact? | Yes…………………… ………  No…………………… ……… | **Close the inquiry** |

**Thank you for your cooperation!!!**
